# Optimal timing of primaquine to reduce *Plasmodium falciparum* gametocyte carriage when co-administered with artemether–lumefantrine

**DOI:** 10.1186/s12936-020-3121-3

**Published:** 2020-01-21

**Authors:** Seif Shekalaghe, Dominic Mosha, Ali Hamad, Thabit A. Mbaga, Michael Mihayo, Teun Bousema, Chris Drakeley, Salim Abdulla

**Affiliations:** 1 Bagamoyo Research and Training Centre (BRTC), Ifakara Health Institute, Bagamoyo, Tanzania; 2 Africa Academy for Public Health, Dar es Salaam, Tanzania; 3 Department of Medical Microbiology, Radboud University Medical Center, Nijmegen, The Netherlands; 4 Department of Immunology and Infection, London School of Hygiene & Tropical Medicine, London, UK

**Keywords:** Malaria, Transmission, Gametocyte, Primaquine, Artemether–lumefantrine

## Abstract

**Background:**

Primaquine is an important gametocytocidal drug that is combined with conventional malaria treatment for prevention of *Plasmodium falciparum* malaria transmission. Primaquine has been administered together on the first or the last day of conventional treatment but the impact of primaquine timing has never been examined. This study aimed to assess safety, efficacy and optimal timing of single full-dose (0.75 mg/kg) primaquine when added to a standard 6-dose regimen of artemether–lumefantrine (AL).

**Methods:**

In an individual-level randomized controlled trial, enrolled participants who were G6PD normal and had uncomplicated *P. falciparum* malaria were randomly assigned to receive: AL only; AL and a single 0.75 mg/kg primaquine dose on the first day of AL (day 1); or AL and single 0.75 mg//kg primaquine on the last day of AL (day 3). On days 2, 3, 4, 8, 11 and 15, gametocytes were assessed and quantified by microscope and quantitative nuclear acid sequence based quantification (QT-NASBA).

**Results:**

Overall, 111 participants aged between 3 and 17 years were randomly allocated to receive AL only (36) or combined with primaquine on day 1 (38), or primaquine on day 3 (37). Day 4 gametocyte prevalence in AL + day 1 primaquine was half the level seen in either AL + day 3 primaquine or AL only arm (11% [4/35] vs 26% [8/31] and 27% [8/30], respectively) albeit not statistically significant. A similar trend of lower gametocyte in the AL + day 1 primaquine verses AL + day 3 primaquine or AL only arm was observed in mean gametocyte density. Mean (sd) haemoglobin level in AL + day 3 primaquine arm recovered from -0.42(1.2) g/dl on day 2 to 0.35 (1.5) g/dl on day 15 of follow up. This was not the case in AL only and AL + day 1 primaquine arms during the same follow-up period, although the difference was not statistically significant (p = 318). No serious adverse events reported in the study. Across arms, 23% (26/111) of participants reported a total of 31 mild adverse events and the difference was not statistically significant (p = 0.477).

**Conclusion:**

Primaquine administration on the first day of AL is well tolerated and as safe as later administration. Whilst the World Health Organization currently recommends a lower dose of primaquine (0.25 mg/kg), the findings are supportive of early primaquine administration when combined with artemisinin-combination therapy.

*ClinicalTrials.gov Registration* NCT01906788

## Background

Malaria elimination is presently considered as a demanding, but achievable objective, even though in 2018 alone there were over 200 million new cases of malaria and 405,000 deaths. Of the deaths, 93% occurred in sub-Saharan Africa [1]. This figure reflects the important malaria burden but also the positive developments witnessed in recent years in the reduction of worldwide burden of the disease [2]. Together with vector control interventions, the use of artemisinin-based combination therapy (ACT) has contributed considerably to the decline in the malaria incidence and prevalence in many sub-Saharan countries [3, 4].

Elimination efforts may benefit from transmission-blocking interventions, including the use of gametocytocidal drugs to prevent *Plasmodium falciparum* transmission and support containment efforts for artemisinin-resistant parasites. The 8-aminoquinoline primaquine, presently represents the only widely available drug option to clear mature *P. falciparum* gametocytes [5, 6]. Until 2012, the World Health Organization (WHO) recommended a single dose of primaquine at 0.75 mg/kg in combination with ACT to treat uncomplicated *P. falciparum* in G6PD normal patients [7]. This has recently been amended to a single low-dose (0.25 mg/kg) of primaquine regardless of the G6PD status, reducing the risk of acute haemolytic reactions in this vulnerable population [8]. However, current policies of some countries such as India and Guyana are still using 0.75 mg/kg dose of primaquine with ACT to treat uncomplicated *P. falciparum* malaria even in G6PD deficient patients [9, 10].

The timing of primaquine when co-administered with ACT may have consequences for primaquine efficacy, safety and compliance [11]. Since ACT effectively clears immature gametocytes, but not mature gametocytes [12], it is conceivable that relatively late dosing of primaquine may be optimal to clear persisting gametocytes. Late primaquine dosing has also been suggested as an approach to reduce haemolytic effects of treatment that are observed after ACT and may be aggravated by primaquine [13, 14]. Additionally, primaquine on the first day of treatment may worsen nausea and vomiting, common malaria presenting symptoms, due to its associated gastrointestinal effects. Patients may better tolerate these symptoms if primaquine is administered on day 3 or later of the treatment [11]. However, administering primaquine on the first day of ACT has operational advantages to ensure compliance because patients can receive the treatment at the clinic once diagnosed with malaria as a directly observed therapy.

The optimal timing of primaquine in combination with ACT in terms of safety and efficacy is relevant for policymakers to decide on the best strategy before wide-scale implementation of ACT-primaquine. The current study thus aimed to determine the optimal timing of a single dose of primaquine administered on either day 1 or 3 in combination with ACT for clearing microscopic and submicroscopic gametocytaemia.

## Methods

### Study area

This study was conducted at Bagamoyo district hospital and Kiwangwa dispensary, Tanzania between May 2013 and July 2015. The trial centre was at Bagamoyo Research and Training Centre (BRTC) of Ifakara Health Institute (IHI) where all laboratory investigations were conducted. Malaria prevalence in the study area is estimated to be 41% by RDT [15]. *Plasmodium falciparum* is the predominant malaria species and the main vector is *Anopheles gambiae* sensu stricto [16].

### Study design

This was a randomized open label clinical trial determining the safety and efficacy of primaquine co-administered at a different time with artemether–lumefantrine (AL) treatment regimen. Recruitment of patients involved screening at two stages. The first screening took place in Bagamoyo district hospital, Kiwangwa dispensary and 4 satellite clinics. This screening selected patients with the following characteristics; aged between 3 and 17 years, parasitaemia of 1000–50,000 parasite/µl, haemoglobin level ≥ 9 g/dl by HemoCue (Angelholm, Sweden), axillary temperature > 37.5 °C and < 39.5 °C or history of fever in the last 48 h, no history of adverse reactions to study medication, no evidence of chronic disease or acute infection other than malaria, not treated with antimalarial chemotherapy in the past 2 weeks, the ability to swallow oral medication, willing to provide written informed consent and comply to study protocol. Participants meeting criteria in satellite clinics were referred to the two study facilities for additional screening and were enrolled after microscopic confirmation for *P. falciparum* mono-infection and when confirmed to be G6PD normal by rapid enzyme chromatographic test (CareStartTM, Access Bio, Inc. New Jersey, USA).

The study was designed to have three treatment groups (1, 2 and 3). Upon meeting all enrolment criteria, participants were randomized to receive either of the three treatment regimens; (i) AL (Coartem; Novartis Pharma AG, Basel Switzerland—20 mg artemether and 120 mg lumefantrine) as a standard six-dose regimen for uncomplicated malaria in Tanzania, (ii) AL and a single 0.75 mg/kg primaquine dose on the first day of AL (day 1 or early primaquine), and (iii) AL and single 0.75 mg/kg primaquine dose on the last day of AL (day 3 or late primaquine). A tablet of 15 mg primaquine (Sanofi Aventis, Mumbai, India) was used. For young children, a tablet(s) adjusted to 0.75 mg/kg dose was crushed and dissolved in 15 mL of clean water and administered orally. All participants were encouraged to eat food before treatment. First dose of AL and primaquine (on day 1 and 3) were given under supervision. Treatment was closely monitored and those vomited within the first 30 min after drug intake were given a second full dose.

### Randomization

Computer generated list of random numbers was used to assign participants into either of the three treatment groups. The sequential list of numbers was matched with treatment group assignments 1, 2 or 3 was prepared and handed to study clinician and was secured in a locked cabinet. Only the study clinician and nurse were aware of treatment assignment. Participants and all other study personnel including field workers and people working in the laboratory were blinded to the treatment assignments.

### Procedures

The treatment was initiated on day 1 (day of enrolment). Participants were asked to return to the study clinic on days 2, 3, 4 8, 11 and 15. On each follow-up day, study participants were assessed for any symptoms, possible adverse events, concomitant medication, measuring axillary temperature and collecting blood samples. In case a participant did not show up at the clinic, a field worker was sent to the house to find out the reason and possibly to bring the participant at the clinic for clinical examination and sampling. At all visits, finger-prick blood samples were collected for blood smears, RNA collection in guanidine isothiocyanate-containing L6 lysis buffer (50 μL of blood in 950 μL buffer) for Pfs25 mRNA quantitative nuclear acid sequence based quantification (QT-NASBA), and haemoglobin level measurements.

Blood slides were stained for 10 min with 10% Giemsa and screened for asexual parasite and gametocytes. Slides were declared negative if no parasites are observed in 100 microscopic fields; asexual parasites and gametocytes counted against 200 and 500 white blood cells, respectively. All slides were examined independently by two experienced technicians using a light microscope. Discrepant findings were reviewed by a third technician until consensus on positivity was reached.

The blood sample collected for QT-NASBA analysis was used to extract RNA first in the field following the original Guanidine isothiocyanate (GuSCN) RNA extraction method [17] and transferred to a laboratory in the Netherlands for completion of the extraction. Nucleic acids were extracted by the Boom upon which Pfs25 mRNA QT-NASBA was performed on a NucliSens EasyQ analyser (bioMérieux, Boxtel, The Netherlands). Gametocyte density was assessed using a trendline of mature stage V gametocytes, as previously reported by Shekalaghe et al. [14].

All clinical and laboratory data were carefully recorded in case record form. Data was double entered into electronic database by experienced data entry clerks. The accuracy of data input was checked and validated using a customized validation program. Data was then converted to Stata dataset for analysis.

### Study end-points

The primary end-point was gametocyte prevalence and density by microscopy and QT-NASBA on day 8, 11 and 15. The secondary end-point was the mean reduction of haemoglobin concentration (g/dl) between baseline and day 4, 8, 11 and 15.

### Sample size

The sample size was decided upon based on an assumed duration of gametocyte carriage by Pfs25 QT-NASBA of 5.2 days (sd = 3.5) with AL-d3 primaquine [14, 18]. It was considered that an increase or reduction in the duration of gametocyte carriage of less than 1.5 days was of limited relevance. With one-sided testing against a p value of 0.05 with 80% power, the authors determined that including 100 individuals at the ratio of 1:1:1 in the three treatment arms would detect differences in the duration of gametocytes carriage of ≥ 1.5 allowing for 10% lost to follow-up. This sample size was not achieved, due to very low inclusion numbers as a result of much lower clinical incidence than anticipated based on historical data.

### Statistical analysis

STATA^®^ 14.0 (Stata Corporation, College Station, Texas, USA) was used for data analysis. Numerical variables were summarized as mean and standard deviation. Categorical variables were summarized using cross tabulation to estimate differences in proportions. The mean time to gametocyte clearance was estimated for each treatment arm using a non-linear model. A 95% confidence interval (CI) was used to determine the difference in gametocyte prevalence between intervention arms (early and late primaquine treatment) and the control arm. Gametocyte prevalence on different visit days was compared between study arms by Chi square or Fisher’s exact test with a two-sided Chi square P-value presented. The area under the curve (AUC) of Pfs25 QT-NASBA gametocyte density versus time was used to determine the effect of treatment. This measure incorporates the duration of gametocyte carriage, from days 0 to 15. A paired t-test was used to compare the changes in haemoglobin level across different time points.

### Ethical consideration

This study was conducted in accordance with the Declaration of Helsinki, ICH Good Clinical Practice, and local regulatory requirements from the Tanzania Food and Drug Authority (TFDA). The study is registered with ClinicalTrials.gov (number NCT01906788). Ethical approval was granted by IHI ethical review board and the National Institute for Medical Research ethical committee (number NIMR/HQ/R.8c/Vol.II/370). Assent and written informed consent was obtained from participants and their parents/guardians, respectively.

## Results

**Fig. 1:**
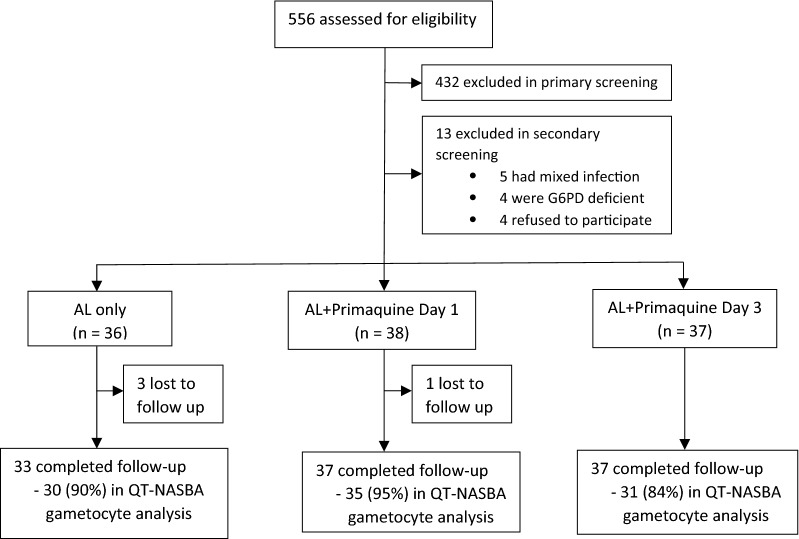
Trial profile

A total of 556 participants were screened for eligibility, 432 (77.7%) and 13 (2.3%) were excluded during primary and secondary screening, respectively. Hundred and eleven (20.0%) were enrolled; 36, 38 and 37 were randomly assigned to AL only, AL + day 1 primaquine and AL + day 3 primaquine arm, respectively. Three participants in AL only arm and 1 in AL + day 3 primaquine were lost during follow up period (Fig. 1). The overall median and interquartile range [IQR] age at enrolment was 7 (5–11) years. Median (IQR) asexual parasite density at baseline was 218 (101–643) parasites/µL, gametocyte density was 3.3 (2.6–3.9) log adjusted gametocytes density/mL and haemoglobin level was 10.6 (9.5–11.6) g/dl (Table 1).

**Table 1:** Baseline characteristics

Characteristics	Treatment arm			p-value
	AL only (n = 33)	AL + primaquine day 1 (n = 37)	AL + primaquine day 3 (n = 37)	
Male sex (%)	17 (51.5)	20 (54.1)	15 (40.5)	0.469
Mean age (SD; range)	8.5 (4.0; 3–17)	7.9 (3.2; 3–16)	7.5 (3.9; 3–17)	0.365
Mean haemoglobin (SD; range)	11.1 (1.2; 9–14)	10.9 (1.6; 9.1–13.8)	10.2 (1.2; 8.7–13.9)	0.441
Mean parasitaemia density (SD; range)	413.3 (596.0; 29–3205)	433.7 (354.7; 28–1116)	349 (317.4; 28–1030)	0.424

*AL* artemether–lumefantrine, *SD* standard deviation

Gametocyte assessment by QT-NASBA, gametocytes were detected up to day 11 for AL only arm (3.3% [1/30]). For AL + day 1 primaquine and AL + day 3 primaquine gametocyte was detected up to day 4 (11.1% [4/35]) and day 8 (6.4% [2/31]), respectively. No gametocytes were detected on day 15 of follow-up in any of the treatment arms. From the baseline, the prevalence of participants with gametocytes steadily decreased in all of the three arms. Day 4 gametocyte prevalence in AL + day 1 primaquine was half the level seen in either AL + day 3 primaquine or AL only arm (4/35 vs 8/31 and 8/30, respectively) although not statistically significant. Day 8 gametocyte prevalence in AL only arm was the highest (13.3% [4/30]) compared to AL + day 1 primaquine (0.0% [0/35]) and + day 3 primaquine (6.4% [2/31]) arm (Tables 2 and 3).

**Table 2:** Asexual and gametocyte prevalence during follow up

	AL only	AL + primaquine day 1	AL + primaquine day 3	P-value	P-value
	% (n/N)	% (n/N)	% (n/N)	(AL only vs AL + primaquine day 1)	(AL only vs AL + primaquine day 3)
Asexual parasite prevalence by microscopy					
Day 2	15.1 (5/33)	24.3 (9/37)	13.5 (5/37)	0.338	0.845
Day 3	0	0	0	–	–

	AL only	AL + primaquine day 1	AL + primaquine day 3		
Gametocyte prevalence by QT-NASBA					
Day 1	90 (27/30)	82.8 (29/35)	96.8 (30/31)	0.474	0.437
Day 4	26.7 (8/30)	11.4 (4/35)	25.8 (8/31)	0.364	0.382
Day 8	13.3 (4/30)	0.0 (0/35)	6.4 (2/31)	–	0.306
Day 11	3.3 (1/30)	0.0 (0/35)	0.0 (0/31)	–	–
Mean AUC (95% CI) of gametocyte density versus time*	0.523 (0.467 – 0.579)	0.530 (0.475 – 0.584)	0.508 (0.450 – 0.563)	0.5319	

*AL* artemether–lumefantrine, *AUC* area under the curve; *CI* confidence interval

*P-value across the three treatment arms

**Table 3:** Day 4 and day 8 gametocyte prevalence adjusted for baseline gametocyte density

Variable	Gametocyte prevalence n/N	OR (95% CI)	p value
Day 4 prevalence			
AL only	8/30	Ref.	
AL + primaquine day 1	4/35	0.28 (0.06 – 1.37)	0.116
AL + primaquine day 3	8/31	0.79 (0.21 – 3.00)	0.726
Day 8 prevalence			
AL only	4/30	Ref.	
AL + primaquine day 1	0/35	–	–
AL + primaquine day 3	2/31	0.51 (0.07 – 3.60)	0.502

*AL* artemether–lumefantrine, *OR* odds ratio, *CI* confidence interval

**Fig. 2:**
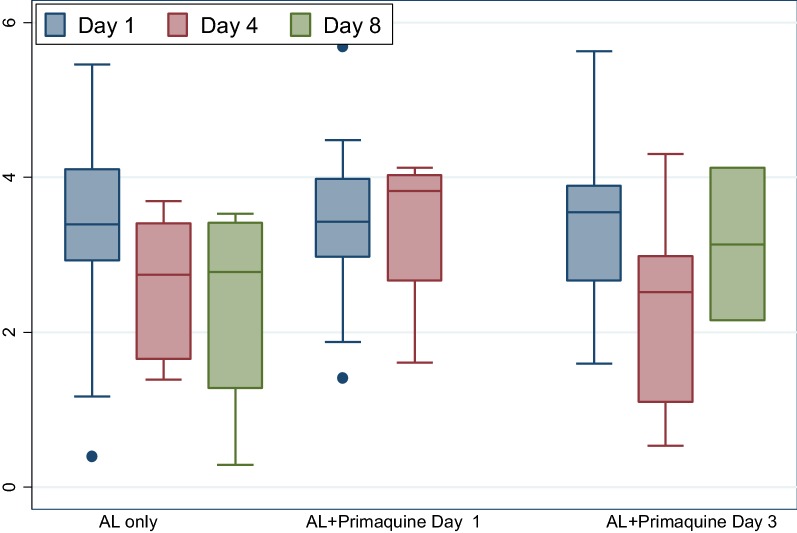
Mean gametocyte concentration measured by QT-NASBA (n = 96)

Mean gametocyte density decreased in all three treatment arms from day 1 to day 4 with a mean difference of 0.754 mL, 0.063 mL, and 1.147 mL in AL only arm, AL + day 1 primaquine and AL + day 3 primaquine, respectively, but the difference across the three arms was not statistically significant., except for AL + day 3 primaquine arm. In the few remaining gametocyte carriers, day 8 mean (sd) gametocyte density in AL + day 3 primaquine arm increased from 2.252 (1.296) mL on day 4 to 3.135 (1.396) mL. Two participants in AL + day 3 primaquine had day 8 mean gametocyte density of 3.135 mL that decreased by 0.539 mL when compared with the density of day 4 (Fig. 2). AUC of gametocyte density versus time was not statistically significant (p = 0.5319) (Table 2).

**Fig. 3:**
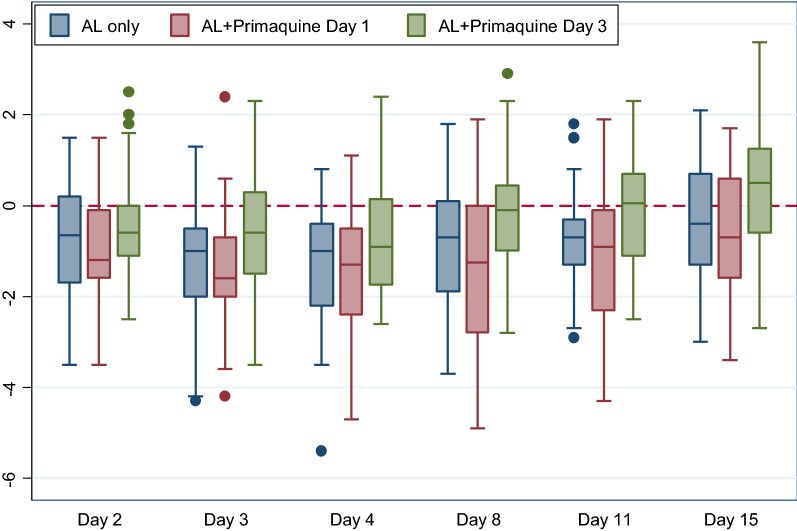
Mean absolute change in haemoglobin concentration per treatment arm (n = 107)

Baseline haemoglobin value was used as a reference to determine relative changes in participant’s haemoglobin level during the follow-up period. The mean (SD) drop of haemoglobin on day 2 was − 0.91 (1.3) g/dl, − 0.92 (1.0) g/dl and − 0.42 (1.2) g/dl in the AL only, AL + day 1 primaquine and AL + day 3 primaquine arms, respectively. Haemoglobin gradually decreased from the baseline values on day 3 and 4 in all of the treatment arms. Day 8 mean drop of haemoglobin was − 0.86 (1.3) g/dl, − 1.26 (1.7) g/dl and − 0.18 (1.2) g/dl for AL only, AL + day 1 primaquine and AL + day 3 primaquine arm, respectively. Haemoglobin level in AL + day 3 primaquine recovered by 0.35 (1.5) g/dl on day 15 of follow up but was not the case in AL only [− 0.39 (1.3)] g/dl and AL + day 1 primaquine [− 0.65 (1.3)] g/dl arms in the same follow up day. The general pattern shows initial sharp decline of haemoglobin level followed by a slow recovery, however, the difference between arms was not statistically significant (p = 318) (Fig. 3).

Twenty-six (23.4%) participants reported a total of 31 mild adverse events; 9 (34.6%) AL only, 6 (23.1) AL + day 1 primaquine and 11 (42.3) AL + day 3 primaquine arm (Table 4). All adverse events were reported in the first 8 days of follow up, 44% of these adverse events were reported on day 2. None of the participants was admitted during the follow-up period as a result of adverse event or for any reason. Cough (34.6%) was the most commonly reported adverse event followed by headache (26.9%). There was no significant difference in terms of proportion or specific adverse events reported between the three treatment arms.

**Table 4:** Self-reported complaints post day 1 of treatment ‘adverse events’

Adverse effects	AL only	AL + primaquine day 1	AL + primaquine day 3
Participants with adverse events (%)	9 (34.6)	6 (23.1)	11 (42.3)
Number of adverse events*	10	8	13
Cough	4	3	2
Skin rush	1	0	0
Headache	2	1	4
Dizziness	1	0	0
Abdominal discomfort/pain	2	2	1
Loss of appetite	0	1	2
Vomiting	0	0	1
Diarrhoea	0	0	1
Otitis media	0	1	0
Conjunctivitis	0	0	1
Painful micturition	0	0	1

*AL* artemether–lumefantrine

*44% of adverse events were reported on day 2 of follow up

Across arms, 23% (26/111) of participants reported a total of 31 mild adverse events; no statistically significant difference in the occurrence of adverse events was observed between arms (p = 0.477).

## Discussion

The study observed that single full-dose primaquine administered with AL on day 1 cleared gametocytes faster than day 3 administration although not statistically significant. Primaquine was well tolerated in both intervention arms with no serious adverse events. No clinically relevant haemolysis was observed; adverse events and reductions in haemoglobin concentration were not significantly different between the control and the two primaquine arms.

Primaquine taken on the first day of AL resulted in the complete absence of gametocytes after the fourth day of treatment initiation as opposed to participants given on the last day of AL in which gametocytes were absent after the eighth day. This suggests that ACT-early primaquine effectively clears both immature and mature gametocytes and makes onward transmission to mosquitoes very unlikely within days after initiation of treatment [11].

Gametocytes in the control arm were detectable until day 11. This contrasts with a similar study conducted in Mali that observed about 90% of patients with gametocytes on day 28 of follow up following AL treatment [19]. The latter study from Mali enrolled high-density gametocyte carriers only; this is the most likely explanation for the observed difference. In addition, AL is superior to dihydroartemisinin–piperiaquine, the ACT used in Mali, in terms of gametocyte reduction. The importance of primaquine for preventing transmission shortly after ACT treatment has been demonstrated for dihydroartemisinin–piperaquine [19, 20] and, with some conflicting findings, for AL [20, 21].

In the current study, mean haemoglobin declined in all of three treatment arms and started to recover from day 7 of treatment. This may be explained by acute haemolysis of parasitized blood cells after treatment with ACT [22]. Previous studies in uncomplicated *P. falciparum* patients have demonstrated similar haemoglobin drops following ACT treatment with or without 0.75 mg/kg or 0.25 mg/kg single dose of primaquine [23–25]. Slow haemoglobin recovery in primaquine day 1 arm may be explained by the effect of primaquine administration on the first day of treatment that may worsen the natural drop in haemoglobin which is typically seen shortly after clearance of malaria parasite [11]. However, there is no significant difference in haemoglobin trend through the follow up period but sample size may have been too small to detect subtle differences.

There were no serious adverse events reported in the study and milder adverse events were often unrelated to treatment and not different between arms.

These results, however, were not statistically significant due to sample size, so the need for further studies should be emphasized, which could use the 0.25 mg/kg WHO recommended primaquine dose instead of 0.75 mg/kg [8], and include adults as well as G6PD deficient patients for whom this dose is considered safer in terms of haemolytic risk. In such study, haemoglobin drops could be followed carefully to address the slower recovery seen in this study for the day 1 primaquine arm (this difference was also not statistically significant in this study).

## Conclusion

The study provided significant information to optimal timing of primaquine in the new WHO recommendation that requires co-administration of ACT with a single low-dose of primaquine (0.25 mg/kg) to patients with *P. falciparum* malaria [8]. Among other reasons, primaquine use on the first day of ACT is equally safe, tolerable and appears superior to late primaquine treatment in terms of gametocyte reduction.

## Data Availability

Not applicable.

## Abbreviations

ACT: artemisinin-based combination therapy
AL: artemether–lumefantrine
BRTC: Bagamoyo Research and Training Centre
G6PD: glucose-6-phosphate dehydrogenase deficiency
IHI: Ifakra Health Institute
QT-NASBA: quantitative nuclear acid sequence based quantification
TFDA: Tanzania Food and Drug Authority
WHO: World Health Organization
